# Are Young Swimmers Short and Middle Distances Energy Cost Sex-Specific?

**DOI:** 10.3389/fphys.2021.796886

**Published:** 2021-12-14

**Authors:** Danilo A. Massini, Tiago A. F. Almeida, Camila M. T. Vasconcelos, Anderson G. Macedo, Mário A. C. Espada, Joana F. Reis, Francisco J. B. Alves, Ricardo J. P. Fernandes, Dalton M. Pessôa Filho

**Affiliations:** ^1^Postgraduate Programme in Human Development and Technologies, São Paulo State University – UNESP, Rio Claro, Brazil; ^2^São Paulo State University – UNESP, Bauru, Brazil; ^3^CIPER, Faculdade de Motricidade Humana, University de Lisboa, Lisbon, Portugal; ^4^School of Education (CIEF – CDP2T), Polytechnic Institute of Setúbal, Setúbal, Portugal; ^5^Quality of Life Research Centre (CIEQV – Politécnico de Leiria), Leiria, Portugal; ^6^Faculdade de Motricidade Humana, Universidade de Lisboa, Lisbon, Portugal; ^7^Faculty of Sport, Centre of Research, Education, Innovation and Intervention in Sport, University of Porto, Porto, Portugal

**Keywords:** oxygen uptake, energy demand, swimming performance, body composition, sex

## Abstract

This study assessed the energy cost in swimming (C) during short and middle distances to analyze the sex-specific responses of C during supramaximal velocity and whether body composition account to the expected differences. Twenty-six swimmers (13 men and 13 women: 16.7 ± 1.9 vs. 15.5 ± 2.8 years old and 70.8 ± 10.6 vs. 55.9 ± 7.0 kg of weight) performed maximal front crawl swimming trials in 50, 100, and 200 m. The oxygen uptake ($\dot{\text{V}}$O_2_) was analyzed along with the tests (and post-exercise) through a portable gas analyser connected to a respiratory snorkel. Blood samples were collected before and after exercise (at the 1st, 3rd, 5th, and 7th min) to determine blood lactate concentration [La^–^]. The lean mass of the trunk (LM_*Trunk*_), upper limb (LM_*UL*_), and lower limb (LM_*LL*_) was assessed using dual X-ray energy absorptiometry. Anaerobic energy demand was calculated from the phosphagen and glycolytic components, with the first corresponding to the fast component of the $\dot{\text{V}}$O_2_ bi-exponential recovery phase and the second from the 2.72 ml × kg^–1^ equivalent for each 1.0 mmol × L^–1^ [La^–^] variation above the baseline value. The aerobic demand was obtained from the integral value of the $\dot{\text{V}}$O_2_ vs. swimming time curve. The C was estimated by the rate between total energy releasing (in Joules) and swimming velocity. The sex effect on C for each swimming trial was verified by the two-way ANOVA (Bonferroni *post hoc* test) and the relationships between LM_*Trunk*_, LM_*UL*_, and LM_*LL*_ to C were tested by Pearson coefficient. The C was higher for men than women in 50 (1.8 ± 0.3 vs. 1.3 ± 0.3 kJ × m^–1^), 100 (1.4 ± 0.1 vs. 1.0 ± 0.2 kJ × m^–1^), and 200 m (1.0 ± 0.2 vs. 0.8 ± 0.1 kJ × m^–1^) with *p* < 0.01 for all comparisons. In addition, C differed between distances for each sex (*p* < 0.01). The regional LM_*Trunk*_ (26.5 ± 3.6 vs. 20.1 ± 2.6 kg), LM_*UL*_ (6.8 ± 1.0 vs. 4.3 ± 0.8 kg), and LM_*LL*_ (20.4 ± 2.6 vs. 13.6 ± 2.5 kg) for men vs. women were significantly correlated to C in 50 (*R*^2^_*adj*_ = 0.73), 100 (*R*^2^_*adj*_ = 0.61), and 200 m (*R*^2^_*adj*_ = 0.60, *p* < 0.01). Therefore, the increase in C with distance is higher for men than women and is determined by the lean mass in trunk and upper and lower limbs independent of the differences in body composition between sexes.

## Introduction

Swimming energy cost (C) expresses the effectiveness of a motor task, allowing the analysis of the motor ability to save or enhance energy production and reflect skilled performance level and muscular work capacity (respectively) from low to high swimming intensities (Fernandes et al., 2006; Zamparo et al., 2008, 2011; Gonjo et al., 2018). In a front crawl, C increases from 0.70 to 1.23 kJ × m^–1^ at 1.0 and 1.5 m × s^–1^, reaching 2.20 kJ × m^–1^ at 2 m × s^–1^ among elite male swimmers (Capelli et al., 1998). The alteration from low to high velocities in swimming requires both muscle power output and energy release to be increased proportionally. Therefore, C defines how mechanical and metabolic capabilities interact to enhance swimming velocity and tolerance according to swimmer sex-group, training status (Toussaint and Hollander, 1994; Capelli et al., 1998; Fernandes et al., 2005, 2006), technical level, and swimming stroke technique (di Prampero et al., 2008; Gonjo et al., 2018).

In elite male swimmers, the energy requirements reach ∼3.33, ∼2.72, and ∼1.94 kW at 45.7, 91.4, and 182.9 m in a front crawl performed at ∼1.97, ∼1.75, and ∼1.62 m × s^–1^ (Capelli et al., 1998). However, the energy requirements attained ∼3.16, ∼1.86, and ∼1.25 kW for young swimmers from both sexes performing 50, 100, and 200 m in a front crawl at ∼1.67, ∼1.46, and ∼1.29 m × s^–1^ (Almeida et al., 2020). These differences in energy contributions and swimming performances would probably rely on the swimmers’ technical and conditioning levels (Fernandes et al., 2006). Muscle mass and fiber composition can also account to those differences, since muscle strength, anaerobic power, and reliance on glycolytic motor units are age group performance influencing factors in short distance swimming races (Hellard et al., 2018).

It is reasonable to consider the amount of muscle mass involved in an exercise with a reliable index of the energetic contribution during a high intensity performance. This is due to how the potential of metabolic resources to the energy releasing can be scaled in body size units, e.g., 0.418 kJ × kg^–1^ for phosphocreatine, 0.0689 kJ × mmol^–1^ × kg^–1^ for blood lactate accumulation, and 0.125 kJ × kg^–1^ for O_2_ stored in arterial blood, i.e., ∼6 ml × kg^–1^ (Medbo et al., 1988). Nevertheless, other key attributes beyond larger muscle mass to anaerobic releasing are greater fast-type muscle fiber composition (enhancing enzymatic lactate dehydrogenase inhibition/activation rulers and redox potential) and glycogen source, which differ between sexes (Esbjörnsson et al., 1993; Esbjörnsson-Liljedahl et al., 1999).

These differences can reflect the advantage in power production by the body region wherein lean mass is larger, e.g., for upper limbs, when comparing men to women (Weber et al., 2006). In swimming, studies corroborating the role of lean mass on high intensity exercise performance have demonstrated that lean mass in upper-limbs correlates with the maximal aerobic velocity, the velocity at 200 m races, and anaerobic reserve estimates among young men (Pessôa Filho et al., 2016). In addition, the highest muscle mass in upper and lower limbs is associated with higher aerobic and anaerobic release during performances lasting 2–3 min among swimmers of both sexes (Ogita et al., 1996). Furthermore, the 400 m front crawl swimming performance peak $\dot{\text{V}}$O_2_ and C differed between prepubertal and pubertal male swimmers, which was a result that can be explained considering the differences in anthropometrical variables, including lean mass (Jürimäe et al., 2007).

However, while adenosine triphosphate turnover requirements of short to middle swimming distances (e.g., 50, 100, and 200 m) are preconized to rely on large anaerobic metabolism demand, with aerobic contribution rising in proportionality to distance-trial length (Almeida et al., 2020), the assumptions for the sex-specific response regarding C and the role of lean mass is lacking. C values for both sexes have been reported for maximal and supramaximal velocities (Zamparo et al., 2000) but the values of C were measured at 1.2, 1.4, and 1.6 m × s^–1^, which were not necessarily velocities corresponding to 50, 100, and 200 m trial performances for all tested swimmers. In addition, the reasons explaining the C differences between sexes at these swimming intensities remain elusive.

Therefore, the association between velocity and energy supply, having sex-based factors as a rule, would evidence a limited rate of energy release for a specific metabolic pathway due to muscle mass difference, even when technical and conditioning levels remain constant. The lack of studies comparing male and female swimmers underappreciated the role of regional and whole-body composition on race performance and swimming training specificity for men and women. Moreover, considering the specific C values during short (50 and 100 m) and middle distances swimming efforts (200 m), the sex differences regarding regional and whole-body lean mass would expect to have an important role. The current study aimed to analyze the C sex-specific responses during supramaximal velocity and if body composition account to the expected differences.

## Materials and Methods

Twenty-six swimmers participated in the current study (13 men and 13 women with 16.7 ± 1.9 vs. 15.5 ± 2.8 years of age, 178.4 ± 8.4 vs. 162.9 ± 7.6 cm of height, 70.8 ± 10.6 vs. 55.9 ± 7.0 kg of weight). All swimmers were regularly engaged in competitive training programs for at least three annual seasons, with a volume of 25 km × week^–1^ during the testing application. Their best front crawl performances at the 50, 100, and 200 m represented 575 ± 95 vs. 534 ± 63, 599 ± 100 vs. 529 ± 78, and 588 ± 94 vs. 552 ± 83 FINA points for male and female swimmers, respectively. Participants were informed about all the study procedures and experimental risks and signed a written informed consent (or their legal guardians when under 18 years old) prior to the experiments. The current research was conducted according to the Declaration of Helsinki and was approved by the Ethics Committee of the São Paulo State University (Protocol 54372516.3.0000.5398).

**FIGURE 1:**
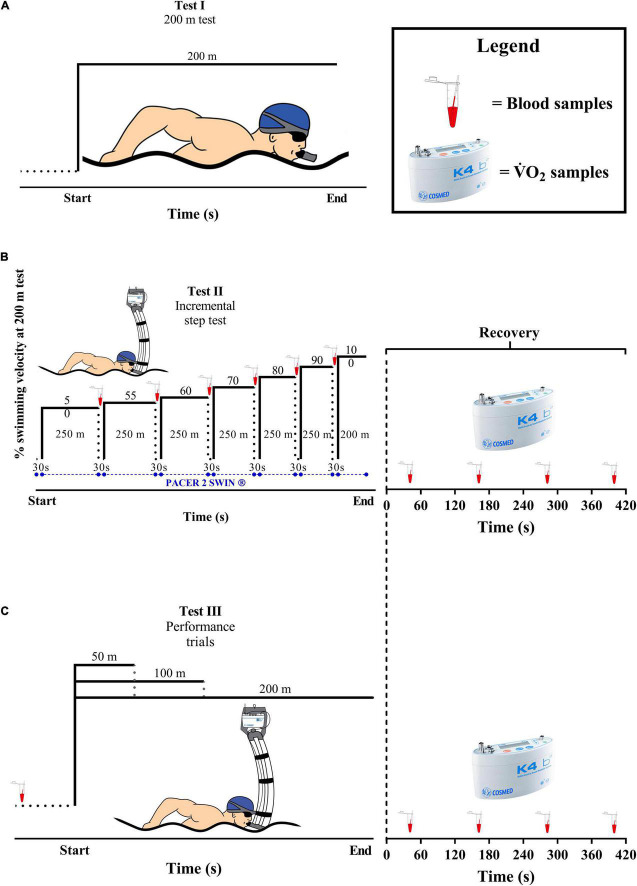
An overview of the experimental protocol. **(A)** The 200 m maximal test. **(B)** The incremental step test. **(C)** 50, 100, and 200 m maximal trials.

The participants performed five tests, all in front crawl and separated by, at least, 24 h: (i) a 200 m maximal test to establish the velocities during the incremental step test; (ii) an incremental step test performed in six progressive steps of 250 m at 50, 55, 60, 70, 80, and 90% plus a single set at 100% of 200 m test, or until voluntary exhaustion (i.e., when swimmers were unable to follow the pacing or stop the exercise (Almeida et al., 2021); and (iii) 50, 100, and 200 m maximal trials (see Figure 1). The control of the swimming velocity during the incremental step test was provided by an underwater LED circuit (Pacer2 Swim^®^, KulzerTEC, Aveiro, Portugal). At the end of each step, a passive rest (30 s) was performed for blood lactate sampling. All procedures were performed in a 25 m indoor pool and, to minimize the differences of prior exercise and the circadian rhythms effects, the same environmental conditions were applied (∼50 of relative humidity, ∼28°C of water temperature, and ±2 h of time of day). The tests were performed during the preparatory period of the training season, and all swimmers went through a familiarization process with the gas collection instruments in the week before the experiments.

Pulmonary gas exchange was analyzed breath-by-breath during and in the 420 s after the incremental step test and the 50, 100, and 200 m maximal trials were analyzed using a portable gas analyzer (K4b^2^, Cosmed, Rome, Italy) connected to the swimmer by a respiratory snorkel (new-AquaTrainer^®^, Cosmed, Rome, Italy; Baldari et al., 2013). The K4b^2^ unit was calibrated before each test according to the manufacturer’s instructions, and the snorkel was connected to the swimmer before each test for assessing the $\dot{\text{V}}$O_2_ baseline (e.g., last 30 s averaged values sampled with swimmer resting for 10 min seated on the pool wall). Blood samples (25 μl) were collected before each test, during the intervals of the incremental step test, and at the 1st, 3rd, 5th, and 7th min after all tests for peak blood lactate concentration determination ([La^–^]_peak_) (YSI, 2300 STAT, Yellow Springs, OH, United States).

Dual-energy x-ray absorptiometry (DXA; Hologic^®^,QDR Discovery Wi^®^) was used for obtaining regional and whole-body composition, with the software Hologic APEX^®^ displaying values for body mass, bone mass, body lean mass (LM_Total_), trunk lean mass (LM_Trunk_), upper limb lean mass (LM_*UL*_), lower limb lean mass (LM_*LL*_), lean mass index (I_*LM*_ = LM × H^1/2^), and appendicular muscle index (I_*App*_ = LM_*App*_ × H ^1/2^). The measurements for upper and lower limbs are the results from the sum of the left and right upper and lower limbs values, respectively, the trunk measurements corresponded to the central body region (from neck to pelvis), and the lean mass measurements result from fat free mass minus bone mineral content (Sala et al., 2007). The equipment was calibrated following the manufacturer’s recommendations, and all the analysis were operated by an experienced technician. Participants wore light clothing and were positioned in the supine position on a flat table with the feet close together and the upper limbs placed parallel to the trunk.

The $\dot{\text{V}}$O_2_ data obtained during incremental step test were smoothed (3 s filter and 15 s moving mean) and peak $\dot{\text{V}}$O_2_ ($\dot{\text{V}}$O_2peak_) considered the highest value observed, while the velocity at the stage where the $\dot{\text{V}}$O_2peak_ was attained and was corresponded to the v$\dot{\text{V}}$O_2peak_, despite the swimmer being able to initiate another step and the $\dot{\text{V}}$O_2_ rise was not larger than ∼2 ml × min^–1^ × kg^–1^ (Reis et al., 2012). From the performance of 50, 100, and 200 m, the breath-by-breath $\dot{\text{V}}$O_2_ was continuously sampled during each trial with a recovery for 420 s. The data were time aligned, followed by noise exclusion (coughing, sighing, and sneezing), which were defined as three standard-deviation from the local mean of five breaths and, finally, the data were interpolated second-by-second (Pessoa Filho et al., 2012). $\dot{\text{V}}$O_2_ off-kinetics was adjusted by a biexponential equation according to Scheuermann et al. (2001) (Eq. 1):


(1) VO2(t)  =EEVO2− A1 off{1−e−[(tf−TD1)τ1]}− A2 off{1−e−[(tf−TD2)τ2]}

where EE$\dot{\text{V}}$O_2_ is the end-exercise $\dot{\text{V}}$O_2_ (the last 15 s moving averaged value), representing the baseline at the very onset of the recovery phase. The physiologically relevant exponential $\dot{\text{V}}$O_2_ response is the primary phase (A_1off_) of the recovery curve and the amplitude of the second phase (A_2off_) corresponds to the slow component of $\dot{\text{V}}$O_2_ recovery (SC$\dot{\text{V}}$O_2_). The time delay (TD_1_ and TD_2_) and time constants (τ_1_ and τ_2_) describe the onset and the velocity of $\dot{\text{V}}$O_2_ recovery in each phase and *t*_*f*_ is the total recovery time. The cardiodynamic phase at the beginning of the recovery was excluded by removing the first 15–20 s of $\dot{\text{V}}$O_2_ response (Özyener et al., 2001).

During each swimming test, the aerobic energy demand (E_Aer_) was obtained from the net $\dot{\text{V}}$O_2_ curve time integral (Eq. 2), and the anaerobic energy demand (E_An_), in O_2_ equivalents (EqO_2_), was obtained by the phosphagen (E_PCr_) and glycolytic (E_[*La*_−_]_) components (Margaria et al., 1933; di Prampero and Ferretti, 1999). The E_PCr_ was determined from the recovery phase fast component ($\dot{\text{V}}$O_2Fast_) using data from the off-kinetic primary phase considering the $\dot{\text{V}}$O_2_ magnitude from the TD_1_ limited to the total recovery time (Stirling et al., 2005; Eq. 3). The amount of 9% corresponding to O_2_ body reserves was subtracted from $\dot{\text{V}}$O_2Fast_ to strictly reflect the E_PCr_ debt after exercise (Medbo et al., 1988; di Prampero and Ferretti, 1999; Weber and Schneider, 2002). E_[*La*_−_]_ was determined according to Eq. 4 (di Prampero and Ferretti, 1999).


(2) $$E_{\text{Aer}}=\int_{t_{0}}^{t_{Lim}}\dot{V}O_{2}\times \text{d}t$$

(3) V˙O2Fast  =A1Off ×τ1{1−e [(tf−TD1)τ1]}+ A1Off{(TD1−tf)e [(tf−TD1)τ1]} 

(4) $$E_{\left[{\text{La}}^{-}\right]}=\left[\left(\beta \times \Delta \left[{\text{La}}^{-1}\right]\times \text{BM}\right)\right]$$

where β is the O_2_ equivalent for each 1.0 mmol × L^–1^ [La^–^] of variation above the baseline value corresponding to 2.72 ml × kg^–1^ in swimming, Δ[La^–^] is the variation of the [La^–^] above the resting value (Δ[La^–^] = [La^–^]_peak_ - [La^–^]_*rest*_), and BM is the whole-body mass in kg.

The estimated absolute values of each of the above-referred energetic components provide total energetic demand (E_Total_) and were converted into J, assuming an energy equivalent of 20.9 kJ × LO_2_^–1^. Subsequently, this energy demand was normalized by the performance time, providing a value in kJ × s^–1^, i.e., the absolute power equivalent. Finally, this power unit was rated by the swimming velocity for each swimming distance providing the C (kJ × m^–1^). The value of the anaerobic C (C_An_) was determined by the sum of C_PCr_ and C_[*La*_−_]_, and the total cost (C_Total_) was obtained from the sum of the C_An_ and aerobic C (C_Aer_).

Normality of the data was checked with Shapiro-Wilk test (*n* < 50), the sphericity by the Mauchly test, and using the Greenhouse-Geisser correction when violated. Independent *t*-student test analyzed the effect of sex on body composition variables and on swimming velocity, time performance, [La^–^]_peak_, and estimated absolute values in EqO_2_, P, and E_Total_ for each of the studied test distances. The differences in energetics and C values between sexes (men vs. women) by distances (i.e., 50, 100, and 200 m) and for each distance by sex were tested by the two-way ANOVA, with Bonferroni as *post hoc* test for pairwise comparison. The effect size for the *t*-student test was calculated using Hedges’ *g* and interpreted as follows: <0.19 (insignificant), 0.20–0.49 (small), 0.50–0.79 (moderate), 0.80–1.29 (large), and >1.30 (very large) (Rosenthal, 1996). For ANOVA, the partial square eta (η^2^_*p*_) was used and interpreted as follows: 0.0099 (small), 0.0588 (medium), and 0.1379 (large; Cohen, 1988).

The relationships between C and body composition variables were assessed by Pearson’s coefficient and classified as follows: 0.00–0.29 (small), 0.30–0.49 (low), 0.50–0.69 (moderate), 0.70–0.89 (high), and 0.90–1.00 (very high; Mukaka, 2012). The regression coefficient that was adjusted to the sample (*R*^2^_*adj*_) analyzed the similarity of variance between C and body composition variables during each 50, 100, and 200 m distance and was considered as <0.04 (trivial), 0.04–0.24 (small), 0.25–0.63 (medium), and >0.64 (strong; Ferguson, 2009). Pearson and regression analysis were controlled for the sex-specific variance of the values. The sample power for the coefficient of correlation, considering the sample size, was the corresponding value of Zα = 1.96 for a security index of α = 0.05. The level of significance was set at ρ ≤ 0.05 for all analysis, with all statistical analyzes performed with the Statistical Package for the Social Sciences (SPSS, version 26.0, Chicago, IL, United States).

## Results

**FIGURE 2:**
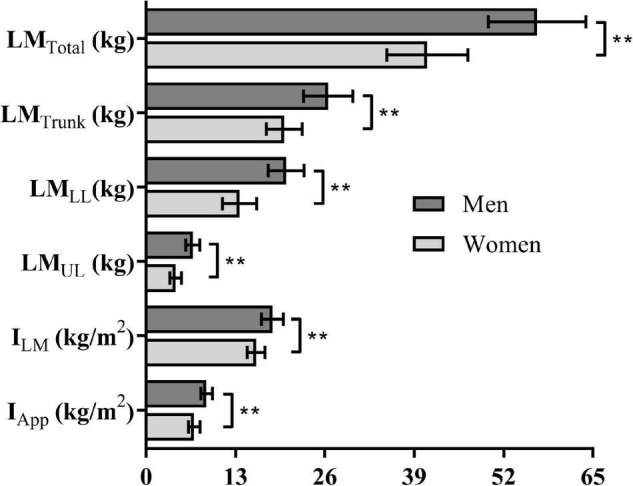
Body composition variables for men and women. Obs.: Significantly different at ρ ≤ 0.01**.

The $\dot{\text{V}}$O_2peak_ values associated to the incremental test was 4.05 ± 0.46 and 3.09 ± 0.36 LO_2_, and v$\dot{\text{V}}$O_2peak_ corresponded to 1.30 ± 0.07 and 1.20 ± 0.06 m × s^–1^, respectively, for men and women. During the v50m, v100m, and v200m, the performances corresponded to 129.8 ± 13.7, 114.8 ± 9.0, and 97.4 ± 7.9% of v$\dot{\text{V}}$O_2peak_ for men and 126.7 ± 8.6, 117.7 ± 6.9, and 101.9 ± 5.7% of v$\dot{\text{V}}$O_2peak_ for women with no differences between sexes for each distance (all at ρ > 0.05). The data related to performances and physiological responses are shown in Table 1. Swimming velocity and *p* were higher for short compared to long swimming distances (i.e., 50 > 100 > 200 m), while total energy measurements in EqO_2_ increased with swimming distance (i.e., 50 < 100 < 200 m). Males demanded higher EqO_2_ and *p* than female swimmers for the 50, 100, and 200 m, but the swimming velocity differed only during 50 and 100 m swimming bouts. Figure 2 highlights the differences between sexes regarding body composition variables. The comparisons between sexes for LM_Total,_ LM_Trunk_, LM_*LL*_, LM_*UL*_, I_*LM*_, and I_*App*_ indicated higher values for men than women (all at ρ < 0.01), with the effect size “*g*” ranging from 1.43 to 2.60 and, therefore, were all considered very large.

**TABLE 1:** Performance and physiological profiles during short and middle distance races.

	Distances (m)		
	50	100	200
Time (s)			
Men	30.0 ± 2.9	67.5 ± 5.3^‡‡^	159.3 ± 12.3^‡‡^^§§^
Women	33.0 ± 0.5**	71.0 ± 3.3^‡‡^	164.1 ± 8.1^‡‡^^§§^
Velocity (m × s^–1^)			
Men	1.68 ± 0.17	1.49 ± 0.12^‡‡^	1.26 ± 0.09^‡‡^^§§^
Women	1.52 ± 0.07**	1.41 ± 0.07*^‡‡^	1.22 ± 0.06^‡‡^^§§^
[La-]_peak_ (mmol × L^–1^)			
Men	9.2 ± 1.9	11.4 ± 2.1^‡‡^	10.2 ± 1.8
Women	9.8 ± 1.4	11.6 ± 1.8^‡‡^	10.9 ± 1.4
Energy, EqO_2_ (L)			
Men	4.13 ± 0.67	6.38 ± 0.77^‡‡^	9.85 ± 1.59^‡‡^^§§^
Women	3.08 ± 0.66**	4.77 ± 0.97**^‡‡^	7.94 ± 1.22**^‡‡^^§§^
Power (kJ × s^–1^)			
Men	2.92 ± 0.64	1.99 ± 0.33^‡‡^	1.30 ± 0.21^‡‡^^§§^
Women	1.96 ± 0.41**	1.41 ± 0.32**^‡‡^	1.01 ± 0.16**^‡‡^^§§^

Significantly different from men at p ≤ 0.01** in 50, 100, and 200 m.

*Significantly different from 50 m at p ≤ 0.01^‡‡^.*

*Significantly different from 100 m at p ≤ 0.01§§.*

**FIGURE 3:**
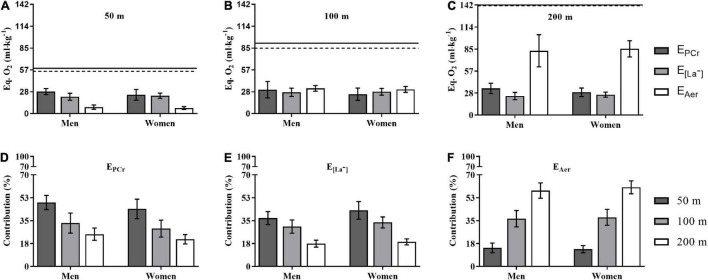
The energetic demand **(A–C)** contribution **(D–F)** to the performance in 50, 100, and 200 m for men and women. Obs.: Horizontal continuous and traced lines at the top of Panels **(A–C)** refer to E_Total_ for men and women, respectively. The acronyms E_PCr_, E_[*La*_−_]_, and E_Aer_ represent the phosphagenic, glycolytic, and aerobic components of E_Total_. See text for statistical analysis.

The E_Total_ demand for men and women during 50 (58.8 ± 8.4 *vs*. 54.9 ± 8.2 ml × kg^–1^), 100 (91.5 ± 14.2 *vs*. 85.0 ± 12.2 ml × kg^–1^), and 200 m (141.9 ± 24.6 *vs*. 141.0 ± 10.4 ml × kg^–1^) did not differ (ρ = 0.24, 0.23, and 0.91) between sexes for each distance. Figure 3 depicts the energetics during the performances of the 50, 100, and 200 m with regards to the demands (Panels A–C, in EqO_2_ per BM) and contributions [Panels D–F, in relative terms (%)] attained by the energetics components (E_PCr_, E_[La–]_, and E_Aer_). The E_PCr_ contribution was higher for men than women only for the 200 m (ρ = 0.04, η^2^p = 0.247), with no differences for the 50 (ρ = 0.75) and 100 m (ρ = 0.13) distances, while the E_[*La*_−_]_ and E_Aer_ components showed no differences between sexes for the 50 (ρ = 0.40 and 0.22), 100 (ρ = 0.73 and 0.37), and 200 m (ρ = 0.30 and 0.70) distances. The contributions of E_PCr_, E_[*La*_−_]_, and E_Aer_ components to the E_Total_ demand in the 50, 100, and 200 m differed between all distances for men and women at ρ < 0.01 level, i.e., %E_PCr_ 50 > 100 > 200 m (η^2^p = 0.815); %E_[*La*_−_]_ 50 > 100 > 200 m (η^2^p = 0.890); and %E_Aer_ 50 < 100 < 200 m (η^2^p = 0.954), whatever the sex. Moreover, men had higher %E_PCr_ in the 200 m than women (ρ = 0.03), while women had higher %E_[*La*_−_]_ in the 50 m than men (ρ = 0.02), with no other differences.

**FIGURE 4:**
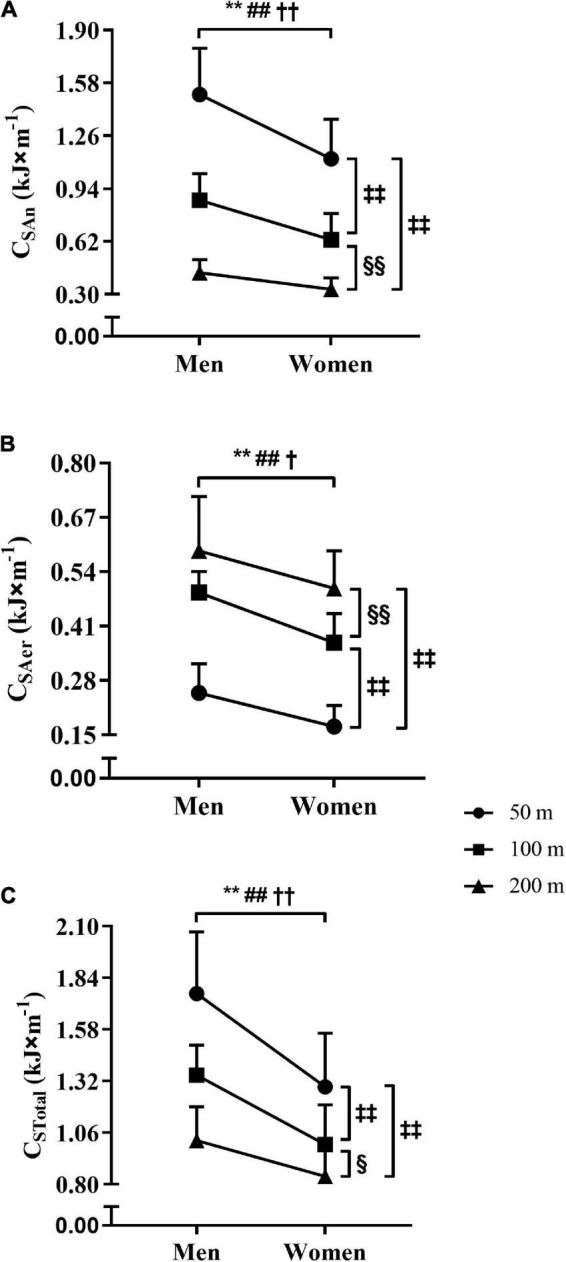
The comparisons of C_An_, C_Aer_, and C_Total_ values **(A–C)** between the sexes and distances. Obs.: The acronyms C_An_, C_Aer_, and C_Total_ represent the anaerobic, aerobic, and total costs. Significantly different from men at *ρ* ≤ 0.05^†^ in 200 m, and at *ρ* ≤ 0.01 in 50**, 100^##^, and 200 m^††^. Significantly different from 50 m at *ρ* ≤ 0.01^‡‡^. Significantly different from 100 m at *ρ* ≤ 0.05 § and ≤ 0.01 §§.

For the performances in the 50, 100, and 200 m tests, the values obtained for C_An_, C_Aer_, and C_Total_ are presented in Figure 4. The Panels A–C (Figure 2) demonstrate the sex-effect on C_An_, C_Aer_, and C_Total_, with higher values for men than women in the 50, 100, and 200 m tests (ρ < 0.01, and η^2^_*p*_ = 0.456, = 0.487, and = 0.519). Also, the reduction of C_An_ and C_Total_ values with the increase of the swimming distance was observed for both sexes (Panels A and C), i.e., C_An_ and C_Total_ in 50 > 100 > 200 m (ρ < 0.01, and η^2^_*p*_ = 0.919 and = 0.778). However, the C_Aer_ values were higher with the increase of the swimming distance for both sexes (Panel B), i.e., C_Aer_ in 50 < 100 < 200 m (ρ < 0.01, and η^2^_*p*_ = 0.838). When expressed per unit of body mass (i.e., J × kg^–1^ × m^–1^), the C_Total_ values did not differ for men vs. women in 50 (24.6 vs. 23.0, ρ = 0.24), 100 (19.1 vs. 17.8, ρ = 0.22), and 200 m (14.8 vs. 14.7, ρ = 0.91).

**FIGURE 5:**
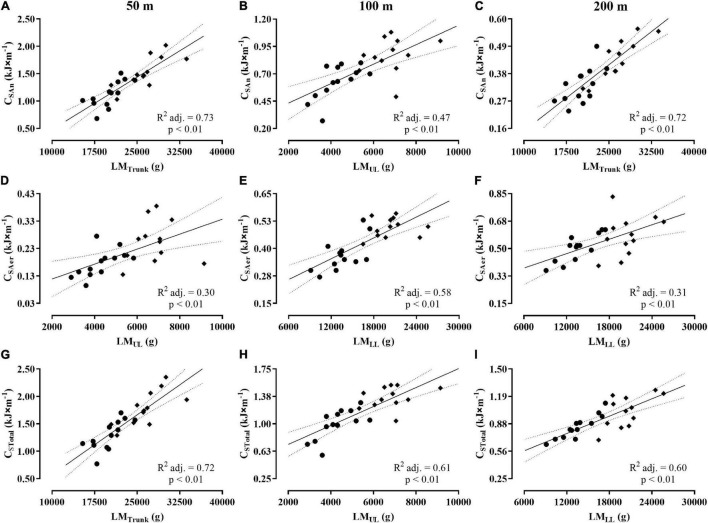
Dispersion plots between body composition variables and values of C_An_, C_Aer_, and C_Total_ for the 50 m **(A,D,G)**, 100 m **(B,E,H)**, and 200 m **(C,F,I)**. Obs.: The symbols (♦) and (●) represent the dispersion for men and women, respectively. The acronyms CAn, CAer, and CTotal represent the anaerobic, aerobic, and total costs.

Table 2 shows the Pearson’s coefficients for the correlations of whole-body and regional lean mass variables with the measurements of C_An_, C_Aer_, and C_Total_ in 50, 100, and 200 m. The correlations were considered significant, classified as moderate or high, and attaining SP ≥ 95% for all analysis, with exception to the LM_*UL*_, I_*LM*_, and I_*App*_ with C_Total_ in the 200 m, which were low and SP < 80%. The variables LM_*Trunk*_, LM_*UL*_, and LM_*LL*_ showed medium to strong influence on C_An_, C_Aer_, and C_Total_ values in the 50, 100, and 200 m (Figure 5, Panels A–I), with LM_*Trunk*_ and LM_*UL*_ attaining high rates to explain the C_An_ values during these distances (Panels A–C), and LM_*LL*_ as the variable explaining the C_Aer_ values for all distances (Panels D–F). Finally, the results at Panels G–I highlight the rates of 72, 61, and 60% for the variables LM_*Trunk*_, LM_*UL*_, and LM_*LL*_, explaining C_Total_ values in 50, 100, and 200 m, respectively.

**TABLE 2:** Relationship between body composition variables and values of C_An_, C_Aer_, and C_Total_ for the 50, 100, and 200 m.

		LM_Total_	LM_Trunk_	LM_LL_	LM_UL_	I_LM_	I_App_
C_An_	50 m	0.85**	0.86**	0.82**	0.81**	0.76**	0.74**
		(100%)	(100%)	(100%)	(100%)	(100%)	(99%)
	100 m	0.68**	0.68**	0.64**	0.70**	0.65**	0.64**
		(97%)	(97%)	(95%)	(98%)	(95%)	(95%)
	200 m	0.85**	0.86**	0.83**	0.82**	0.77**	0.75**
		(100%)	(100%)	(100%)	(100%)	(100%)	(100%)
C_Aer_	50 m	0.56**	0.54**	0.56**	0.57**	0.47*	0.52**
		(84%)	(82%)	(84%)	(86%)	(67%)	(76%)
	100 m	0.75**	0.71**	0.73**	0.73**	0.65**	0.71**
		(100%)	(99%)	(100%)	(99%)	(95%)	(99%)
	200 m	0.56**	0.54**	0.59**	0.46*	0.41*	0.45*
		(84%)	(82%)	(88%)	(66%)	(53%)	(63%)
C_Total_	50 m	0.85**	0.86**	0.83**	0.82**	0.75**	0.75**
		(100%)	(100%)	(100%)	(100%)	(100%)	(100%)
	100 m	0.78**	0.77**	0.76**	0.79**	0.73**	0.74**
		(100%)	(100%)	(100%)	(100%)	(99%)	(99%)
	200 m	0.78**	0.78**	0.79**	0.71**	0.65**	0.66**
		(100%)	(100%)	(100%)	(98%)	(95%)	(96%)

*Obs.: Data are showing the coefficient (r) and sample power in percentage.*

Significantly different at ρ ≤ 0.05* and ≤0.01**.

*LM_Total,_ LM_Trunk_, LM_LL_, and LM_UL_ are lean mass in whole-body, trunk and lower and upper limbs, and I_LM_ and I_App_ are lean mass index and appendicular lean mass index.*

## Discussion

This study addressed the C during short and middle distances performances in swimming, finding a sex-specific response regarding the energetics contribution to the performances, to C during each swimming distance, and to the role of regional lean mass on C values. The findings indicated no differences between sexes for the E_Total_ and for the components E_PCr_, E_[*La*_−_]_, and E_Aer_, suggesting similar capacity between young men and women to meet the energy requirements per unit of body mass in a front crawl. However, the C_Total_ was higher in men than women for all swimming distances performed, despite how both sexes presented similar C components regarding the reliance on C_*Ana*_ and C_Aer_ expenditure, respectively, during short distances (50 and 100 m) and middle distances (200 m). For the current study, these differences in C_An_, C_Aer_, and C_Total_ can be attributed to the increased production of metabolic power in men, which was observed to relate to lean mass in the trunk and upper and lower limbs.

The similarities for E_PCr_, E_[*La*_−_]_, and E_Aer_ in the 50, 100, and 200 m (with E_PCr_ at the 200 m being the only exception) support the evidence that fast-energy pathways (i.e., phosphagens and glycolysis), level of activation, and contribution, while the oxidative supply is rising from short to middle distances performances, have no constraints related to sex-specific energy metabolism. In addition, similarities were also noted to the interplay (% of contribution) between E_PCr_, E_[*La*_−_]_, and E_Aer_ as trial time increases from the 50 to 200 m, evidencing that sex has no influence on given metabolism requirements neither on the balance between the metabolism components as the demand changes according to the swimming intensity and duration over the distances.

These findings are aligned with the evidence toward similarities in energetics rely on fiber type distribution in biceps brachialis and vastus lateralis, with no differences between young men and women, and on the reports relating fiber firing to exercise intensity as sex-independent (Esbjörnsson et al., 1993; Miller et al., 1993; Hunter, 2016). In addition, other reports evidencing larger fiber areas (I, IIa, and IIb) of the biceps brachialis and vastus lateralis in men than women (Esbjörnsson et al., 1993; Miller et al., 1993) probably account for the differences in total muscle PCr content between sexes (Esbjörnsson et al., 1993; Esbjörnsson-Liljedahl et al., 1999). Therefore, the PCr content might explain the small differences between sexes observed in the current study and account for the higher reliance on E_PCr_ in men compared to women as the distance increases from 50 to 200 m, and for the women reliance on larger E_[*La*_−_]_ than men during the performance of short distance, i.e., 50 m.

However, the finding in which no differences between men and women, regarding anaerobic glycolytic contribution, is not in agreement with the well-reported reduced glycolytic activity for women when compared to men during Wingate and MAOD tests in cycling and running (Esbjörnsson et al., 1993; Gratas-Delamarche et al., 1994; Naughton et al., 1997; Hill and Vingren, 2011). These studies attributed the differences upon glycolytic demand to the higher absolute exercise intensity reached by men (i.e., peak power) since no significant differences between sexes in power (Maud and Shultz, 1986; Nindl et al., 1995; Hegge et al., 2015) or anaerobic demand (Weber and Schneider, 2002) were found when whole-body and regional mass or lean mass were considered.

In addition, the [La^–^]_peak_ values for the current study are aligned with the values reported for the 50, 100, and 200 m maximal swimming performances (Chatard et al., 1988; Troup et al., 1992; Capelli et al., 1998) and, therefore, the acidosis level is compatible to the other results exploring the energetics requirements during short and middle distances swimming performance. Furthermore, the observed similarities between men vs. women in [La^–^]_peak_ and E_[*La*_−_]_ responses cannot be attributed to the trial differences in %v$\dot{\text{V}}$O_2peak_ and duration during each performance since these parameters were not significantly different between sexes. The only exception was the duration of 50 m, which was smaller in men than women. However, the differences seem to not be large enough to modify glycolytic contribution according to sex-specific performances.

The lack of differences between sexes was also observed for E_Aer_ responses in 50, 100, and 200 m, which was an expected result considering the limited capacity to uptake and deliver oxygen to the working muscles at exercise rates higher than or close to $\dot{\text{V}}$O_2peak_ for women is associated to the body size differences to men and, therefore, relating energetics to a scaling issue (Weber and Schneider, 2002). Indeed, the absolute differences in cardiac, circulatory, and respiratory determinants of O_2_ availability to muscle while exercising near or at 100% $\dot{\text{V}}$O_2peak_ are not significant when comparing sexes per unit of lean mass (Peltonen et al., 2013), which are further supported to the evidence that fiber type I functions and distribution, pulmonary diffusive capacity, blood volume, and hemoglobin content are not different when comparing between sexes accounting to the effect of body size or lean mass (Esbjörnsson-Liljedahl et al., 1999; Russ et al., 2005; Haizlip et al., 2015; Bouwsema et al., 2017; Koons et al., 2019).

Analogous to the evidence of “size” effect on energetics between sexes (Chatard et al., 1991), the results from the current study also observed higher absolute energy demand in EqO_2_ for men compared to women in the 50, 100, and 200 m tests, either considering the total (i.e., E_Total_) or the components of the energetics (i.e., E_PCr_, E_[*La*_−_]_ and E_Aer_). These results remain unchanged even when considering the performance variable time or velocity to normalize energy demand, giving important insights into the relevance of body morphology for the sex-specific energetics response per unit of time or distance while swimming at high intensity rates. The remarkable finding is the predominance of anaerobic energy releasing per unit of distance in the 50 and 100 m, whereas aerobic energy predominated along the 200 m. Furthermore, aerobic and anaerobic C differed between sexes, with lean mass in the trunk and upper and lower limbs explaining 60–73% of C_Total_ for 50–200 m.

In swimming, the energy contribution from phosphagen, glycolytic, and aerobic components during 50, 100, and 200 m has been reported to differ between sexes only in 200 m and with regards the phosphagen (men > women) and glycolytic (men < women) contributions (Almeida et al., 2020). When comparing the current data from the energy released with the aforementioned reports, there are slight differences with regards to anaerobic energetics, which are probably due to the methodological assumptions for the estimation of phosphagen and glycolytic components (i.e., the subtracted amount of hemoglobin O_2_ content and the stoichiometric coefficient for blood lactate net accumulation – Medbo et al., 1988; di Prampero and Ferretti, 1999) since swimmers are similar with regards to age group, conditioning index ($\dot{\text{V}}$O_2peak_ and v$\dot{\text{V}}$O_2peak_), and performance pacing (v and %v$\dot{\text{V}}$O_2peak_). However, the differences between the studies are also large for aerobic contribution, which just reinforce the concerns on the data processing strategies influence on $\dot{\text{V}}$O_2_ analysis (Robergs et al., 2010). However, the interpretation from Almeida et al. (2020) that energetics during short and middle distances performance did not differ between sexes per unit of body mass is aligned with the current results.

Indeed, the reports for aerobic and anaerobic percentage of contribution to total energy released by elite male swimmers in 50 (∼15/85%), 100 (∼33/77%), and 200 yards (∼62/38%) (Capelli et al., 1998) are quite aligned to the current findings and therefore supports the reliance on anaerobic sources during performances around 60 s, which has already been demonstrated for swimming (Capelli et al., 1998; Ogita et al., 2003) and cycling exercise (Bangsbo et al., 1990; Spencer and Gastin, 2001). The current results indicated a larger phosphagen than glycolytic contribution for the anaerobic releasing during 50 and 200 m in men, and similar contributions for these two components in women during 50, 100, and 200 m, which are not aligned to previous reports. In fact, highest glycolytic reliance during short and middle distances performances have been observed in elite male swimmers [i.e.: E_PCr_/E_[*La*_−_]_ (%) ∼26/54, 19/43, 13/24 in 50, 100 and 200 yards, Capelli et al., 1998; and E_PCr_/E_[*La*_−_]_ (%) ∼11/15 in 200 m, Sousa et al., 2013] and for junior and senior male swimmers [i.e., E_PCr_/E_[*La*_−_]_ (%) ∼20/27 and 18/37 in 100 m, Hellard et al., 2018], but exceptionally, Figueiredo et al. (2011) reported E_PCr_/E_[*La*_−_]_ (%) ∼20/14% in 200 m, which is close to the proportionality in the current study.

The aforementioned estimates of E_PCr_, supposing a maximal depletion of PCr store (i.e., 18.5 mmol/kg of wet muscle at 23.4 s time constant for substrate splitting), have been suggested as reasonable (Capelli et al., 1998; Hellard et al., 2018) and expected to give similar results when compared to the analyzes of the fast component of $\dot{\text{V}}$O_2_ recovery curve (at least for the 200 m swimming performance; Sousa et al., 2013). However, in the current study, the values observed for the time constant of the $\dot{\text{V}}$O_2_ recovery fast component ranged from 44 to 46 s for 50, 100, and 200 m, which are in the range reported for severe exercise in cycling (35 ± 11 s), high intensity lower limbs extension (51 ± 6 s) (Özyener et al., 2001; Rossiter et al., 2002), and short distance swimming trial (Almeida et al., 2020) but diverge with that reported for 200 m in swimming (27 ± 5 s, Sousa et al., 2013). Despite the differences of parameters selection (i.e., EE$\dot{\text{V}}$O_2_ *vs*. $\dot{\text{V}}$O_2baseline_) for the equation model that can account for the differences of the time constant response, the evidence of similarities or discrepancies between methods for phosphagen component estimation needed to be further investigated.

Nevertheless, considering that the release of ∼3.33, ∼2.72, and ∼1.94 kW in elite male college swimmers during short and middle distances performances at ∼139, ∼123, and ∼114% v$\dot{\text{V}}$O_2max_ (Capelli et al., 1998), and that the maximal anaerobic supply during high-intensity performances can reach 1,452 J × kg^–1^ (or ∼69.5 ml × kg^–1^) in well-trained swimmers (Toussaint and Hollander, 1994), we suppose that the swimmers in the current study are still in the development training stage therefore requiring metabolic power output and anaerobic capacity improvements. Despite how the women have shown lower values, the average anaerobic release (i.e., E_PCr_ + E_[*La*_−_]_) reached the highest values during the 100 and 200 m (e.g., ∼57 ml × kg^–1^) which is lower than values for the 200 m (∼68 ml × kg^–1^) reported in international level male swimmers (Fernandes et al., 2006), corroborating the range for improvements in the aforementioned variables for young swimmers. However, the current values are also revealed to be higher than anaerobic release during 100 m (e.g., ∼48 and 54 ml × kg^–1^) as reported for male swimmers with 18–22 years old and low to high FINA points (Hellard et al., 2018), and higher than the estimated anaerobic capacity (e.g., ∼50–52 ml × kg^–1^) for college swimmers (Ogita et al., 1996, 2003; Capelli et al., 1998).

From these comparisons, there is considerable support to consider no sex-constraints among young swimmers to reach the expecting anaerobic conditioning to compete at a high level despite the transference for elite performance being limited to the fact that current swimmers are well-trained but not top-level athletes. However, the energy releasing sources do not seem to be the only determinant to the performance level during short and middle distances (Figueiredo et al., 2013; Ribeiro et al., 2017; Zacca et al., 2020), although the variables power and cost have been considered determinants of swimming performance, exercise tolerance, total energy requirement, and aerobic/anaerobic metabolism balance during high-intensity bouts (Toussaint, 1990; Chatard et al., 1991; Fernandes et al., 2006; di Prampero et al., 2008).

For example, as swimming velocity increases, the metabolic power should raise proportionality to afford mechanical adjustments with no technical impairments (i.e., accommodating higher stroke rate with minimal disturbance in stroke length), allowing to differentiate swimmers according to the conditioning and technical levels (Toussaint, 1990; Wakayoshi et al., 1995; Ribeiro et al., 2017). This explains the lower race pace, energy power, and cost when comparing men from the current study with college male swimmers performing short and middle distances, or even the economical pacing of these later swimmers when compared to the ones from the current study by estimating C from front crawl equation (=0.228[10^.488v^], Capelli et al., 1998) at the same average velocity in 50, 100, and 200 m (e.g., ∼1.5 vs. ∼1.7; ∼1.2 vs. ∼1.3; and ∼.9 vs. ∼1.0 kJ × m^–1^). Despite that the economy is a feature of the skilled technique, other variables like age, anthropometry, training level, and engaged muscle mass can account for C difference among male swimmers (Chatard et al., 1990, 1991; Fernandes et al., 2006; Morris et al., 2016; Hellard et al., 2018), which seems to be the case for the comparisons with values from the current study.

However, the current findings are aligned with the statements on the C augmentation with swimming front crawl velocity increment at supramaximal velocities (Capelli et al., 1998), which was observed for both sexes. The increase in C with velocity has been demonstrated for young female swimmers with a different level of performance in 400 m, while performing a common range of velocities below each group level from v400m (Chatard et al., 1991), for teenage women during the performance of 50, 100, 200, and 400 m (Zamparo et al., 2000), and between young competitive female swimmers performing 200 m with different stroke rate values (Morris et al., 2016). Although the C values for female swimmers are scarce for performances at supramaximal velocities, a single study demonstrated that young women spent 19, 15, and 10% less energy when compared to young men at 1.2, 1.4, and 1.6 m × s^–1^ (Zamparo et al., 2000), which were not, necessarily, the actual velocities for 400, 200, 100, and 50 m. Therefore, the current findings can be useful to compare C measurement methods and analyze performance levels while swimming at actual 50, 100, and 200 m events.

For example, the average C values reported for high ranked young female swimmers at 1.4 m × s^–1^ (or ∼103% v$\dot{\text{V}}$O_2peak_) was 27.3 ml × m^–1^ (Unnithan et al., 2009), which is 31% lower than the C estimated in the current study at the correspondent swimming intensity (∼102% v$\dot{\text{V}}$O_2peak_ at 200 m) or 43% lower than C at the same pacing (∼1.41 m × s^–1^ at 100 m). Taking into account that these authors assessed only $\dot{\text{V}}$O_2_ response to estimate C, and that anaerobic contribution to 200 and 100 m can reach ∼29 and ∼46%, respectively, for women (Almeida et al., 2020), these C values can be considered equivalent. Indeed, the C values for women observed in the current study for 200 m are only ∼8% higher than the C for low trained level female swimmers (∼13.6 J × kg^–1^ × m^–1^) performing at 1.2 m × s^–1^ (or ∼103% v$\dot{\text{V}}$O_2peak_), but are 25% higher than C of high-trained level female swimmers (∼11.7 J × kg^–1^ × m^–1^) performing at the same absolute pacing (1.2 m × s^–1^) but at lower relative intensity (∼86% v$\dot{\text{V}}$O_2peak_) (Fernandes et al., 2006). While the comparison with low-trained swimmers did not differ, since the E_PCr_ was not considered to the energetics measurements, which usually account for more than ∼10% at exercise rate (Sousa et al., 2013), the comparison to the high-trained woman highlights the importance of swimming economy to the athlete consolidation.

Moreover, the current findings also observed that the differences in C_Total_ between sexes during each distance were eliminated when expressed in body mass units, which is aligned to the reports for both sexes at the same absolute submaximal pacing (i.e., 1.3 m × s^–1^) but different exercise rate for men *vs*. women: ∼90 *vs*. ∼98% v$\dot{\text{V}}$O_2peak_ (Fernandes et al., 2006). However, previous studies comparing both sexes at 100% v$\dot{\text{V}}$O_2peak_ (Fernandes et al., 2005) or at different stroke rates and velocities (Morris et al., 2016) found higher C for men than women, which was considered an effect of high velocity or stroke rate achieved at $\dot{\text{V}}$O_2peak_ in men and, therefore, different energy requirement compared to women. The current findings support that the higher C for young men than young women while performing 50 to 200 m can be attributed to the highest velocity performed by men, which is probably accounted to the larger hydrodynamic resistance (Zamparo et al., 2000, 2008).

The current study did not observe differences in C when scaled to the body mass, which may be occurred due to the paired supramaximal exercise rate where hydrodynamics compromises both sexes maximally and hence accounting less to explain the C values variation with velocity (Zamparo et al., 2000, 2008). Also, differences between sexes of C values at swimming circumstance >100%v$\dot{\text{V}}$O_2peak_, lasting 30–150 s, would be supported to the differences in $\dot{\text{V}}$O_2_ adjustments to its maximum and the rate of anaerobic stores depletion, which have been theoretically demonstrated by comparing swimmers while swimming with different stroke technique or having no similar $\dot{\text{V}}$O_2peak_ level (di Prampero et al., 2008). In absence of this case, the technical proficiency (favoring women) and the energetic releasing (favoring men) would be balanced by a given similar C between sexes. However, this still remains in a theoretical scenery and could be explored in the future studies by analyzing swimmers with similar C.

Finally, this is the first study demonstrating that swimmers with the largest lean mass in the trunk and upper limb are less economical while performing 50 and 100 m because lean mass is related to high anaerobic C, and swimmers with the largest lean mass in the lower limb should present more aerobic C, whatever the sex. On the other hand, if C increases with swimming velocity demanding high metabolic energy (Zamparo et al., 2000, 2008; di Prampero et al., 2008), then lean mass content between swimmers is crucial to the improvement of short and middle distance performances, which is a sex-specific C statement complementing that reporting body mass and composition as explanatory variables for energy metabolism and performance differences between athletes from different maturation level (Hellard et al., 2018).

Inasmuch as the biological level of maturation for each sex-group was not determined in the current study, we are unable to refute the fact that maturation level has an effect on energetics and C, and on the relation of these variables with lean mass. Thus, this is a limitation of the current findings, indicating that the interplay between lean mass and energy releasing could be an effect of maturation and not related to sex differences (Jürimäe et al., 2007) or, at least, suggesting limited transference to other age-groups. Although, swimmers were supposed to have similar status respective to each sexual developmental stage, as suggested to the low variability of lean mass, height, and body weight values in each sex-group (Zemel, 2013).

Furthermore, as traditional or specific resistance training can modify force-velocity relationship in muscle and neuromuscular coordination affecting swimming performance positively along with increasing lean mass (Crowley et al., 2017), it should therefore be highly recommended to explore in future studies the potential of muscle hypertrophy to improve swimming performance during supramaximal exercise rates. Taking all of these in consideration, the findings suggest young male and female swimmers can improve their actual conditioning level, and, therefore, their short and middle distances performances by following exercises planning to improve trunk and upper and lower limbs lean mass, enabling limbs muscles to attend for high C demands.

## Conclusion

The current study observed sex independence on the profile of contribution and reliance of the energetics components during high intensity swimming performance. This evidence is favoring no constraints for the energetics capability of women to match men’s energy balance and releasing during high intensity swimming performance. Moreover, current results about C are aligned to the notion that differences between sexes on energetics are related to body mass and composition, and therefore eliminated when scaled to body size dimensions. However, this finding refers to an analysis not encompassing top-level athletes, but concern to swimmers in-preparation and with similar training experience and conditioning levels for which the differences in hydrodynamics and supramaximal exercise rates are minor. Finally, the specificities of each sex regarding the energetics and lean mass responses to training should be further explored in future studies engaging top-level swimmers from different age-groups.

## Data Availability Statement

The raw data supporting the conclusions of this article will be made available by the authors, without undue reservation.

## Ethics Statement

The studies involving human participants were reviewed and approved by Ethics Committee of the São Paulo State University (Protocol 54372516.3.0000.5398). Written informed consent to participate in this study was provided by the participants’ legal guardian/next of kin.

## Author Contributions

DM, TA, JR, FA, and DP conceived and designed the study. DM, TA, CV, ME, AM, JR, and DP conducted the experiments and analyzed the data. DM, TA, ME, AM, JR, FA, RF, and DP wrote the manuscript. All authors contributed to the article, read, and approved the manuscript.

## Conflict of Interest

The authors declare that the research was conducted in the absence of any commercial or financial relationships that could be construed as a potential conflict of interest.

## Publisher’s Note

All claims expressed in this article are solely those of the authors and do not necessarily represent those of their affiliated organizations, or those of the publisher, the editors and the reviewers. Any product that may be evaluated in this article, or claim that may be made by its manufacturer, is not guaranteed or endorsed by the publisher.

## References

[B1] Almeida T. A. F. Pessôa Filho D. M. Espada M. A. C. Reis J. F. Sancassani A. Massini D. A. (2021). Physiological responses during high-intensity interval training in young swimmers. *Front. Physiol.* 12:662029. 10.3389/fphys.2021.662029 34276394 PMC8281220

[B2] Almeida T. A. F. Pessôa Filho D. M. Espada M. A. C. Reis J. F. Simionato A. R. Siqueira L. O. C. (2020). $\dot{\text{V}}$O2 kinetics and energy contribution in simulated maximal performance during short and middle distance-trials in swimming. *Eur. J. Appl. Physiol.* 120 1097–1109. 10.1007/s00421-020-04348-y 32212025

[B3] Baldari C. Fernandes R. J. Meucci M. Ribeiro J. Vilas-Boas J. P. Guidetti L. (2013). Is the new aquatrainer^®^ snorkel valid for $\dot{\text{V}}$O2 assessment in swimming? *Int. J. Sports Med.* 34 336–344. 10.1055/s-0032-1321804 23041962

[B4] Bangsbo J. Gollnick P. D. Graham T. E. Juel C. Kiens B. Mizuno M. (1990). Anaerobic energy production and O2 deficit-debt relationship during exhaustive exercise in humans. *J. Physiol.* 422 539–559. 10.1113/jphysiol.1990.sp018000 2352192 PMC1190148

[B5] Bouwsema M. M. Tedjasaputra V. Stickland M. K. (2017). Are there sex differences in the capillary blood volume and diffusing capacity response to exercise? *J. Appl. Physiol.* 122 460–469. 10.1152/japplphysiol.00389.2016 27932673 PMC5401957

[B6] Capelli C. Pendergast D. R. Termin B. (1998). Energetics of swimming at maximal speeds in humans. *J. Appl. Physiol.* 78 385–393. 10.1007/s004210050435 9809837

[B7] Chatard J. C. Lavoie J. M. Lacour J. R. (1991). Energy cost of front crawl swimming in women. *Eur. J. Appl. Physiol.* 63 12–16. 10.1007/BF00760794 1915325

[B8] Chatard J. C. Lavoie J. M. Lacourl J. R. (1990). Analysis of determinants of swimming economy in front crawl. *Eur. J. Appl. Physiol.* 61 88–92. 10.1007/BF00236699 2289503

[B9] Chatard J. C. Paulin M. Lacour J. R. (1988). “Postcompetition blood lactate measurements and swimming performance: illustrated by data from a 400 m olympic record holder,” in *Swimming Science V*, eds Ungerechts B. E. Wilke K. Reischle K. (Champaign, IL: Human Kinetics), 311–316.

[B10] Cohen J. (ed.) (1988). *Statistical Power Analysis for the Behavioral Sciences.* Hillsdale, NJ: L. Erlbaum Associates, 10.4324/9780203771587

[B11] Crowley E. Harrison A. J. Lyons M. (2017). The impact of resistance training on swimming performance: a systematic review. *Sports Med.* 47 2285–2307. 10.1007/s40279-017-0730-2 28497283

[B12] di Prampero P. E. Dekerle J. Capelli C. Zamparo P. (2008). The critical velocity in swimming. *Eur. J. Appl. Physiol.* 102 165–171. 10.1007/s00421-007-0569-6 17901978

[B13] di Prampero P. E. Ferretti G. (1999). The energetics of anaerobic muscle metabolism: a reappraisal of older and recent concept. *Respir. Physiol.* 118 103–115. 10.1016/s0034-5687(99)00083-3 10647856

[B14] Esbjörnsson M. Sylven C. Holm I. Jansson E. (1993). Fast twitch fibres may predict anaerobic performance in both females and males. *Int. J. Sports Med.* 14 257–263. 10.1055/s-2007-1021174 8365833

[B15] Esbjörnsson-Liljedahl M. Sundberg C. J. Norman B. Jansson E. (1999). Metabolic response in type I and type II muscle fibers during a 30-s cycle sprint in men and women. *J. Appl. Physiol.* 87 1326–1332. 10.1152/jappl.1999.87.4.1326 10517759

[B16] Ferguson C. J. (2009). An Effect size primer: a guide for clinicians and researchers. *Prof. Psychol. Res. Pract.* 40 532–538. 10.1037/a0015808

[B17] Fernandes R. Billat V. Cruz A. Colaço P. Cardoso C. Vilas-Boas J. P. (2005). Has gender any effect on the relationship between time limit at VO2max velocity and swimming economy? *J. Hum. Mov. Stud.* 49 127–148.

[B18] Fernandes R. J. Billat V. L. Cruz A. C. Colaço P. J. Cardoso C. S. Vilas-Boas J. P. (2006). Does net energy cost of swimming affect time to exhaustion at the individual’s maximal oxygen consumption velocity? *J. Sports Med. Phys. Fitness* 46 373–380. 16998440

[B19] Figueiredo P. Pendergast D. R. Vilas-Boas J. P. Fernandes R. J. (2013). Interplay of biomechanical, energetic, coordinative, and muscular factors in a 200 m front crawl swim. *Biomed. Res. Int.* 2013:897232. 10.1155/2013/897232 23586063 PMC3613086

[B20] Figueiredo P. Zamparo P. Sousa A. Vilas-Boas J. P. Fernandes R. J. (2011). An energy balance of the 200 m front crawl race. *Eur. J. Appl. Physiol.* 111 767–777. 10.1007/s00421-010-1696-z 20978781

[B21] Gonjo T. McCabe C. Sousa A. Ribeiro J. Fernandes R. J. Vilas-Boas J. P. (2018). Differences in kinematics and energy cost between front crawl and backstroke below the anaerobic threshold. *Eur. J. Appl. Physiol.* 118 1107–1118. 10.1007/s00421-018-3841-z 29556773

[B22] Gratas-Delamarche A. Le Cam R. Delamarche P. Monnier M. Koubi H. (1994). Lactate and catecholamine responses in male and female sprinters during a Wingate test. *Eur. J. Appl. Physiol. Occup. Physiol.* 68 362–366. 10.1007/BF00571458 8055897

[B23] Haizlip K. M. Harrison B. C. Leinwand L. A. (2015). Sex-based differences in skeletal muscle kinetics and fiber-type composition. *Physiol* 30 30–39. 10.1152/physiol.00024.2014 25559153 PMC4285578

[B24] Hegge A. M. Myhre K. Welde B. Holmberg H. C. SandbakK O. (2015). Are gender differences in upper-body power generated by elite cross-country skiers augmented by increasing the intensity of exercise? *PLoS One.* 10:e0127509. 10.1371/journal.pone.0127509 26000713 PMC4441444

[B25] Hellard P. Pla R. Rodríguez F. A. Simbana D. Pyne D. B. (2018). Dynamics of the metabolic response during a competitive 100 m freestyle in elite male swimmers. *Int. J. Sports. Physiol. Perform.* 13 1011–1020. 10.1123/ijspp.2017-0597 29466071

[B26] Hill D. W. Vingren J. L. (2011). Maximal accumulated oxygen deficit in running and cycling. *Appl. Physiol. Nutr. Metab.* 36 831–838. 10.1139/h11-108 22050108

[B27] Hunter S. K. (2016). The relevance of sex differences in performance fatigability. *Med. Sci. Sports Exerc.* 48 2247–2256. 10.1249/MSS.0000000000000928 27015385 PMC5349856

[B28] Jürimäe J. Haljaste K. Cicchella A. Lätt E. Purge P. Leppik A. (2007). Analysis of swimming performance from physical, physiological, and biomechanical parameters in young swimmers. *Pediatr. Exerc. Sci.* 19 70–81. 10.1123/pes.19.1.70 17554159

[B29] Koons N. J. Suresh M. R. Schlotman T. E. Convertino V. A. (2019). Interrelationship between sex, age, blood volume, and $\dot{\text{V}}$O2max. *Aerosp. Med. Hum. Perform.* 90 362–368. 10.3357/AMHP.5255.2019 30922423

[B30] Margaria R. Edwards H. T. Dill D. B. (1933). The possible mechanisms of contracting and paying the oxygen debt and the role of lactic acid in muscular contraction. *Am. J. Physiol.* 106 689–714. 10.1152/ajplegacy.1933.106.3.689

[B31] Maud P. J. Shultz B. B. (1986). Gender comparisons in anaerobic power and anaerobic capacity tests. *Br. J. Sports Med.* 20 51–54. 10.1136/bjsm.20.2.51 3730753 PMC1478312

[B32] Medbo J. I. Mohn A. C. Tabata I. Bahr R. Vaage O. Sejersted O. M. (1988). Anaerobic capacity determined by maximal accumulated O2 deficit. *J. Appl. Physiol.* 64 50–60. 10.1152/jappl.1988.64.1.50 3356666

[B33] Miller A. E. J. Macdougall J. D. Tarnopolsky M. A. Sale D. G. (1993). Gender differences in strength and muscle fiber characteristics. *Eur. J. Appl. Physiol. Occup. Physiol.* 66 254–262. 10.1007/bf00235103 8477683

[B34] Morris K. S. Osborne M. A. Shephard M. E. Skinner T. L. Jenkins D. G. (2016). Velocity, aerobic power and metabolic cost of whole body and arms only front crawl swimming at various stroke rates. *Eur. J. Appl. Physiol.* 116 1075–1085. 10.1007/s00421-016-3372-4 27052972

[B35] Mukaka M. M. (2012). Statistics corner: a guide to appropriate use of correlation coefficient in medical research. *Malawi Med. J.* 24 69–71. 23638278 PMC3576830

[B36] Naughton G. A. Carlson J. S. Buttifant D. C. Selig S. E. Meldrum K. Mckenna M. J. (1997). Accumulated oxygen deficit measurements during and after high-intensity exercise in trained male and female adolescents. *Eur. J. Appl. Physiol. Occup. Physiol.* 76 525–531. 10.1007/s004210050285 9404864

[B37] Nindl B. C. Mahar M. T. Harman E. A. Patton J. F. (1995). Lower and upper body anaerobic performance in male and female adolescent athletes. *Med. Sci. Sports Exerc.* 27 235–241. 7723647

[B38] Ogita F. Hara M. Tabata I. (1996). Anaerobic capacity and maximal oxygen uptake during arm stroke, leg kicking and whole-body swimming. *Acta. Physiol. Scand.* 157 435–441. 10.1046/j.1365-201X.1996.490237000.x 8869726

[B39] Ogita F. Onodera T. Tamaki H. Toussaint H. M. Hollander A. P. Wakayoshi K. (2003). “Metabolic profile during exhaustive arm stroke, leg kick, and whole-body swimming lasting 15s to 10 min,” in *Biomechanics and Medicine in Swimming IX*, ed. Chatard J. C. (Saint-Etienne: University of Saint-Etienne), 361–366.

[B40] Özyener F. Rossiter H. B. Ward S. A. Whipp B. J. (2001). Influence of exercise intensity on the on-and off-transient kinetics of pulmonary oxygen uptake in humans. *J. Physiol.* 533 891–902. 11410644 10.1111/j.1469-7793.2001.t01-1-00891.x PMC2278649

[B41] Peltonen J. E. Hägglund H. Koskela-Koivisto T. Koponen A. S. Aho J. M. Rissanen A. P. E. (2013). Alveolar gas exchange, oxygen delivery and tissue deoxygenation in men and women during incremental exercise. *Respir. Physiol. Neurobiol.* 188 102–112. 10.1016/j.resp.2013.05.014 23707876

[B42] Pessoa Filho D. M. Alves F. B. Reis J. F. Greco C. C. Denadai B. S. (2012). $\dot{\text{V}}$O2 kinetics during heavy and severe exercise in swimming. *Int. J. Sports Med.* 33 744–748. 10.1055/s-0031-1299753 22592546

[B43] Pessôa Filho D. M. Simionato A. R. Siqueira L. O. D. C. Espada M. A. Pestana D. (2016). Influence of regional and whole-body composition on swimming performance and aerobic indices. *Braz J of Sports Med* 22 195–199. 10.1590/1517-869220162203151766

[B44] Reis J. F. Alves F. B. Bruno P. M. Vleck V. Millet G. P. (2012). Oxygen uptake kinetics and middle-distance swimming performance. *J. Sci. Med. Sport.* 15 58–63. 10.1016/j.jsams.2011.05.012 21802360

[B45] Ribeiro J. Toubekis A. G. Figueiredo P. de Jesus K. Toussaint H. M. Alves F. (2017). Biophysical determinants of front crawl swimming at moderate and severe intensities. *Int. J. Sports Physiol. Perform.* 12 241–246. 10.1123/ijspp.2015-0766 27248207

[B46] Robergs R. A. Dwyer D. Astorino T. (2010). Recommendations for improved data processing from expired gas analysis indirect calorimetry. *Sports Med.* 40 95–111. 10.2165/11319670-000000000-00000 20092364

[B47] Rosenthal J. A. (1996). Qualitative descriptors of strength of association and effect size. *J. Soc. Service Res.* 21 37–59. 10.1300/J079V21N04_02

[B48] Rossiter H. B. Ward S. A. Kowalchuk J. M. Howe F. A. Griffiths J. R. Whipp B. J. (2002). Dynamic asymmetry of phosphocreatine concentration and O2 uptake between the on-and off-transients of moderate-and high-intensity exercise in humans. *J. Physiol.* 541 991–1002. 10.1113/jphysiol.2001.012910 12068057 PMC2290368

[B49] Russ D. W. Lanza I. R. Rothman D. Kent-Braun J. A. (2005). Sex differences in glycolysis during brief, intense isometric contractions. *Muscle Nerve* 32 647–655. 10.1002/mus.20396 16025523

[B50] Sala A. Webber C. E. Morrison J. Beaumont L. F. Barr R. D. (2007). Whole-body bone mineral content, lean body mass, and fat mass measured by dual-energy X ray absorptiometry in a population of normal Canadian children and adolescents. *Can. Assoc. Radiol. J.* 58 46–52. 17408162

[B51] Scheuermann B. W. Hoelting B. D. Noble M. L. Barstow T. J. (2001). The slow component of O2 uptake is not accompanied by changes in muscle EMG during repeated bouts of heavy exercise in humans. *J. Physiol.* 531 245–256. 10.1111/j.1469-7793.2001.0245j.x 11179407 PMC2278436

[B52] Sousa A. Figueiredo P. Zamparo P. Vilas-Boas J. P. Fernandes R. J. (2013). Anaerobic alactic energy assessment in middle distance swimming. *Eur. J. Appl. Physiol.* 113 2153–2158. 10.1007/s00421-013-2646-3 23609331

[B53] Spencer M. R. Gastin P. B. (2001). Energy system contribution during 200-to 1500 m running in highly trained athletes. *Med. Sci. Sports Exerc.* 33 157–162. 10.1097/00005768-200101000-00024 11194103

[B54] Stirling J. R. Zakynthinaki M. S. Saltin B. (2005). A model of oxygen uptake kinetics in response to exercise: including a means of calculating oxygen demand/deficit/debt. *Bull. Math. Biol.* 67 989–1015. 10.1016/j.bulm.2004.12.005 15998492

[B55] Toussaint H. M. (1990). Differences in propelling efficiency between competitive and triathlon swimmers. *Med. Sci. Sports Exerc.* 22 409–415. 2381311

[B56] Toussaint H. M. Hollander A. P. (1994). Energetics of competitive swimming: implications for training programs. *Exerc. Sports Sci. Rev.* 18 384–405. 10.2165/00007256-199418060-00004 7886354

[B57] Troup J. Hollander A. Bone M. Trappe S. Barzdukas A. (1992). “Performance-related differenced in the anaerobic contribution of competitive freestyle swimmers,” in *Biomechanics and Medicine in Swimming VI*, eds MacLaren D. Reilly T. Lees A. (London: E & FN SPON), 271–278.

[B58] Unnithan V. Holohan J. Fernhall B. Wylegala J. Rowland T. Pendergast D. R. (2009). Aerobic cost in elite female adolescent swimmers. *Int. J. Sports Med.* 30 194–199. 10.1055/s-0028-1104583 19199194

[B59] Wakayoshi K. D’Acquisto L. Cappaert J. Troup J. (1995). Relationship between oxygen uptake, stroke rate and swimming velocity in competitive swimming. *Int. J. Sports Med.* 16 19–23. 10.1055/s-2007-972957 7713625

[B60] Weber C. L. Chia M. Inbar O. (2006). Gender differences in anaerobic power of the arms and legs–a scaling issue. *Med. Sci. Sports Exerc.* 38 129–137. 10.1249/01.mss.0000179902.31527.2c 16394965

[B61] Weber C. L. Schneider D. A. (2002). Increases in maximal accumulated oxygen deficit after high-intensity interval training are not gender dependent. *J. Appl. Physiol.* 92 1795–1801. 10.1152/japplphysiol.00546.2001 11960926

[B62] Zacca R. Azevedo R. Ramos V. R. Jr. Abraldes J. A. Vilas-Boas J. P. de Souza Castro F. A. (2020). Biophysical follow-up of age-group swimmers during a traditional three-peak preparation program. *J. Strength Cond. Res.* 34 2585–2595. 10.1519/JSC.0000000000002964 30640304

[B63] Zamparo P. Capelli C. Cautero M. Di Nino A. (2000). Energy cost of front crawl swimming at supramaximal speeds and underwater torque in young swimmers. *Eur. J. Appl. Physiol.* 83 487–491. 10.1007/s004210000318 11192054

[B64] Zamparo P. Capelli C. Pendergast D. (2011). Energetics of swimming: a historical perspective. *Eur. J. Appl. Physiol.* 111 367–378. 10.1007/s00421-010-1433-7 20428884

[B65] Zamparo P. Lazzer S. Antoniazzi C. Cedolin S. Avon R. Lesa C. (2008). The interplay between propelling efficiency, hydrodynamic position and energy cost of front crawl in 8 to 19-year-old swimmers. *Eur. J. Appl. Physiol.* 104 689–699. 10.1007/s00421-008-0822-7 18636269

[B66] Zemel B. (2013). Bone mineral accretion and its relationship to growth, sexual maturation and body composition during childhood and adolescence. *World Rev. Nutr. Diet.* 106 39–45. 10.1159/000342601 23428679

